# The small Ca^2+^-binding protein CSE links Ca^2+^ signalling with nitrogen metabolism and filament integrity in *Anabaena* sp. PCC 7120

**DOI:** 10.1186/s12866-020-01735-5

**Published:** 2020-03-11

**Authors:** Julia Walter, Francisco Leganés, Eva-Mari Aro, Peter J. Gollan

**Affiliations:** 1 Department of Biochemistry, Molecular Plant Biology, University of Turku, Tykistökatu 6A, 6. krs, 20520 Turku, Finland; 2 Present address: Department of Plant Sciences, Environmental Plant Physiology, University of Cambridge, Downing Street, Cambridge, CB2 3EA UK; 3 Departamento de Biología, Facultad de Ciencias, Universidad Autónoma de Madrid, Calle Darwin 2, 28049 Madrid, Spain

**Keywords:** Anabaena, Calcium, Cyanobacteria, Heterocysts, Nitrogen fixation, Filaments

## Abstract

**Background:**

Filamentous cyanobacteria represent model organisms for investigating multicellularity. For many species, nitrogen-fixing heterocysts are formed from photosynthetic vegetative cells under nitrogen limitation. Intracellular Ca^2+^ has been implicated in the highly regulated process of heterocyst differentiation but its role remains unclear. Ca^2+^ is known to operate more broadly in metabolic signalling in cyanobacteria, although the signalling mechanisms are virtually unknown. A Ca^2+^-binding protein called the Ca^2+^ Sensor EF-hand (CSE) is found almost exclusively in filamentous cyanobacteria. Expression of *asr1131* encoding the CSE protein in *Anabaena* sp. PCC 7120 was strongly induced by low CO_2_ conditions, and rapidly downregulated during nitrogen step-down. A previous study suggests a role for CSE and Ca^2+^ in regulation of photosynthetic activity in response to changes in carbon and nitrogen availability.

**Results:**

In the current study, a mutant *Anabaena* sp. PCC 7120 strain lacking *asr1131* (*Δcse*) was highly prone to filament fragmentation, leading to a striking phenotype of very short filaments and poor growth under nitrogen-depleted conditions. Transcriptomics analysis under nitrogen-replete conditions revealed that genes involved in heterocyst differentiation and function were downregulated in *Δcse*, while heterocyst inhibitors were upregulated, compared to the wild-type.

**Conclusions:**

These results indicate that CSE is required for filament integrity and for proper differentiation and function of heterocysts upon changes in the cellular carbon/nitrogen balance. A role for CSE in transmitting Ca^2+^ signals during the first response to changes in metabolic homeostasis is discussed.

## Background

In cyanobacteria, the ancestors of plant chloroplasts, the role of calcium ions (Ca^2+^) in abiotic stress response was identified by the recombinant expression of bioluminescent Ca^2+^ sensors. A transient increase in the intracellular Ca^2+^ concentration ([Ca^2+^]_i_) from a resting level of about 0.2 μM up to 4 μM free Ca^2+^ was observed upon light-to-dark transitions and during temperature, salt and osmotic stress [1–3]. The involvement of Ca^2+^ has also been established in cyanobacterial growth [4], regulation of reactive oxygen species (ROS) [5], fine-tuning of the carbon (C) and nitrogen (N) balance [6, 7], heat stress acclimation [8], exopolysaccharide production [9], and fatty acid and hydrocarbon composition [10].

According to their morphology, cyanobacteria are classified into unicellular and multicellular (filamentous) species [11]. The filamentous *Nostoc* sp. PCC 7120 (hereafter designated *Anabaena*) represents a group of organisms of special interest because of their ability to fix atmospheric N under combined N-limiting growth conditions, which occurs within specialised cells called heterocysts (reviewed in [12]). A prominent role of Ca^2+^ in *Anabaena* is in the regulation of heterocyst formation [13], which is thought to occur through the activity of the cyanobacterial Ca^2+^-binding protein (CcbP), which binds Ca^2+^ via negative surface charges [14, 15]. In N-limiting conditions, CcbP was reportedly strongly downregulated, both at the expression level by the transcriptional regulator NtcA, and at the protein level through HetR-mediated proteolysis. The decrease in CcbP abundance resulted in an increase in free Ca^2+^ in differentiating cells 5 to 6 h after removal of combined N [16]. HetR also acts as a transcription factor, which regulates expression of several genes involved in the commitment of a vegetative cell to differentiation into a proheterocyst and maturation into a functional heterocyst. This genetic reprogramming includes inhibition of cell division and formation of the heterocyst envelope, comprising a gas-impermeable glycolipid layer and outer polysaccharide layer [17–19]. This developmental process occurs in about every tenth cell of a filament under N-deprived conditions, due to the action of heterocyst pattern formation proteins such as the small peptide PatS, which is expressed mainly in heterocysts [20, 21] and diffuses into adjacent cells where it inhibits the activity of HetR [22].

Proper heterocyst development in *Anabaena* is closely coupled with filament integrity. Several mutant strains of *Anabaena* that are defective in heterocyst differentiation and function exhibit a fragmented filament phenotype upon N deprivation [23–31]. Proteins required for both filament integrity and heterocyst development in the absence of combined N include cell envelope components [31], in particular the SepJ (also called FraG) protein, which is localised to the septum structure between cells, as well as a series of other “Fra” proteins [25, 28, 32–34]. Recently the gene cluster *fraCDEF* was identified, from which the corresponding proteins FraCDE promote filament elongation, whereas FraF restricts filament length [29]. FraCD and SepJ were shown to be involved in the formation of septum-localised channels for communication, and for exchange of reduced C (sugars) and combined N metabolites (amino acids) between CO_2_-fixing vegetative cells and N-fixing heterocysts. Mutants lacking these proteins fragmented and became unviable upon the shift to N-deficient media due to the malformation of septal structures [35–37].

We recently identified the Ca^2+^-binding protein Ca^2+^ Sensor EF-hand (CSE) to be highly conserved in filamentous, heterocystous cyanobacteria [7]. Here we describe mutant strains of *Anabaena* lacking CSE, which demonstrated severe filament fragmentation and were impaired in heterocyst formation and function. We propose a role for CSE in transducing the Ca^2+^ signal required for early heterocyst differentiation, which implicates Ca^2+^ and CSE in responding to and restoring the C/N balance in N-fixing cyanobacteria.

## Results

### Deletion of CSE leads to filament fragmentation and compromised N-fixing ability

**Fig. 1:**
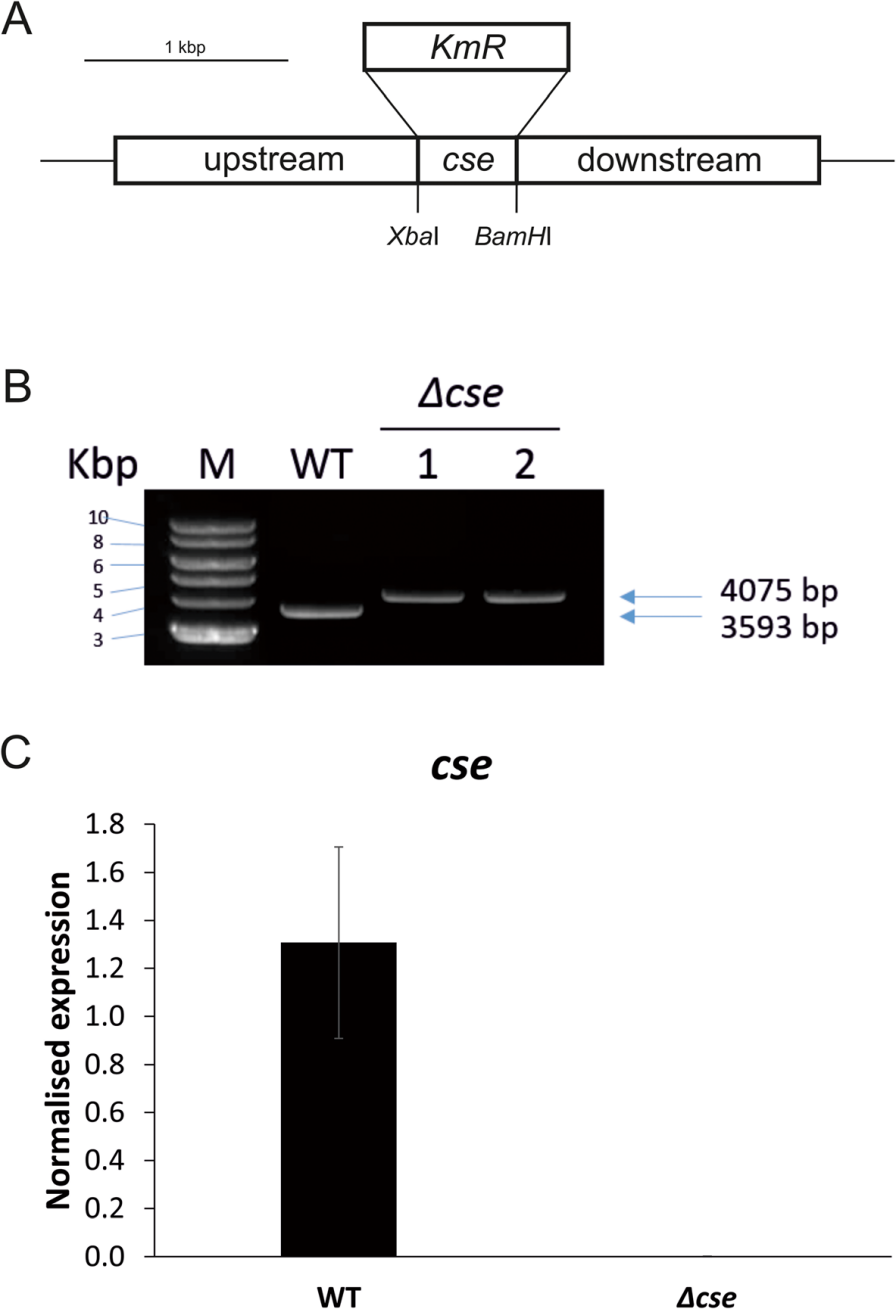
Construction and verification of the *Δcse* mutant. **a** Scheme of the *Δcse* vector construct, with a kanamycin/neomycin resistance cassette (KmR/NmR) replacing the *cse* gene. **b** Polymerase chain reaction (PCR) confirmation of two independently-obtained, fully segregated *Δcse* clones. Amplification of the *cse* gene with its upstream and downstream flanking regions in *Anabaena* wild-type (WT) resulted in a PCR product of 3593 bp. In the *Δcse* clones, replacement of *cse* with KmR/NmR resulted in a larger PCR product of 4075 bp. No traces of the WT PCR product in the *Δcse* clones indicated full segregation of the mutant. **c** Expression of *cse* in WT and *Δcse* clone 1 normalised to the expression of the reference gene *rpoA*. Error bars indicate the standard deviation from three biological replicates (*n* = 3)

**Fig. 2:**
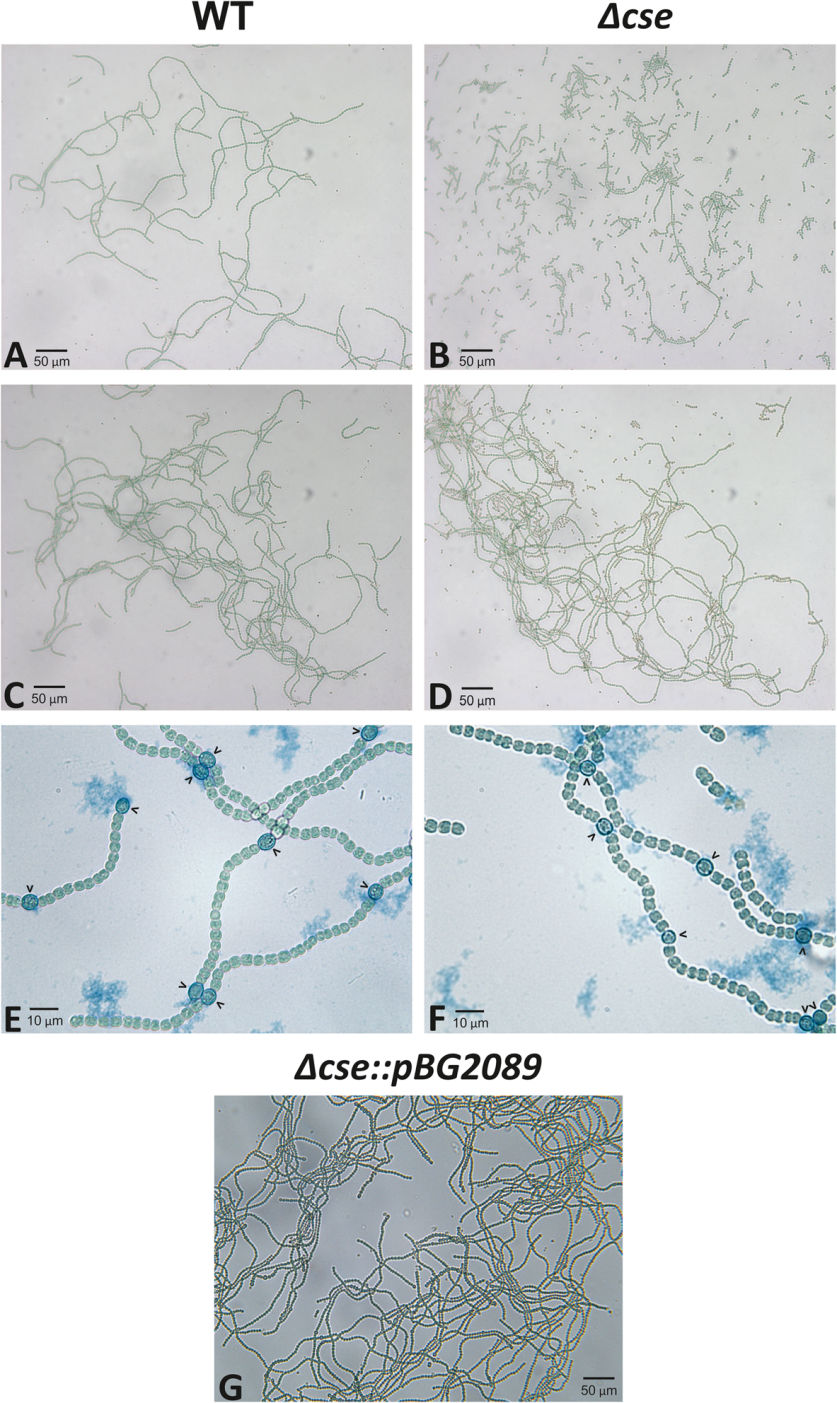
Phenotype of the *Δcse* mutant. Bright-field micrographs of four-day old cultures of wild-type (WT; left), *Δcse* (right) and *Δcse::pBG2089* (**g**) growing in 3% CO_2_ in regular BG11 medium (**a**, **b**, **g**) or in BG11_0_ medium lacking combined nitrogen (**c** and **d**). Alcian Blue stains (**e** and **f**) were used to visualise heterocysts and proheterocysts, indicated by carets, in long filaments

**Fig. 3:**
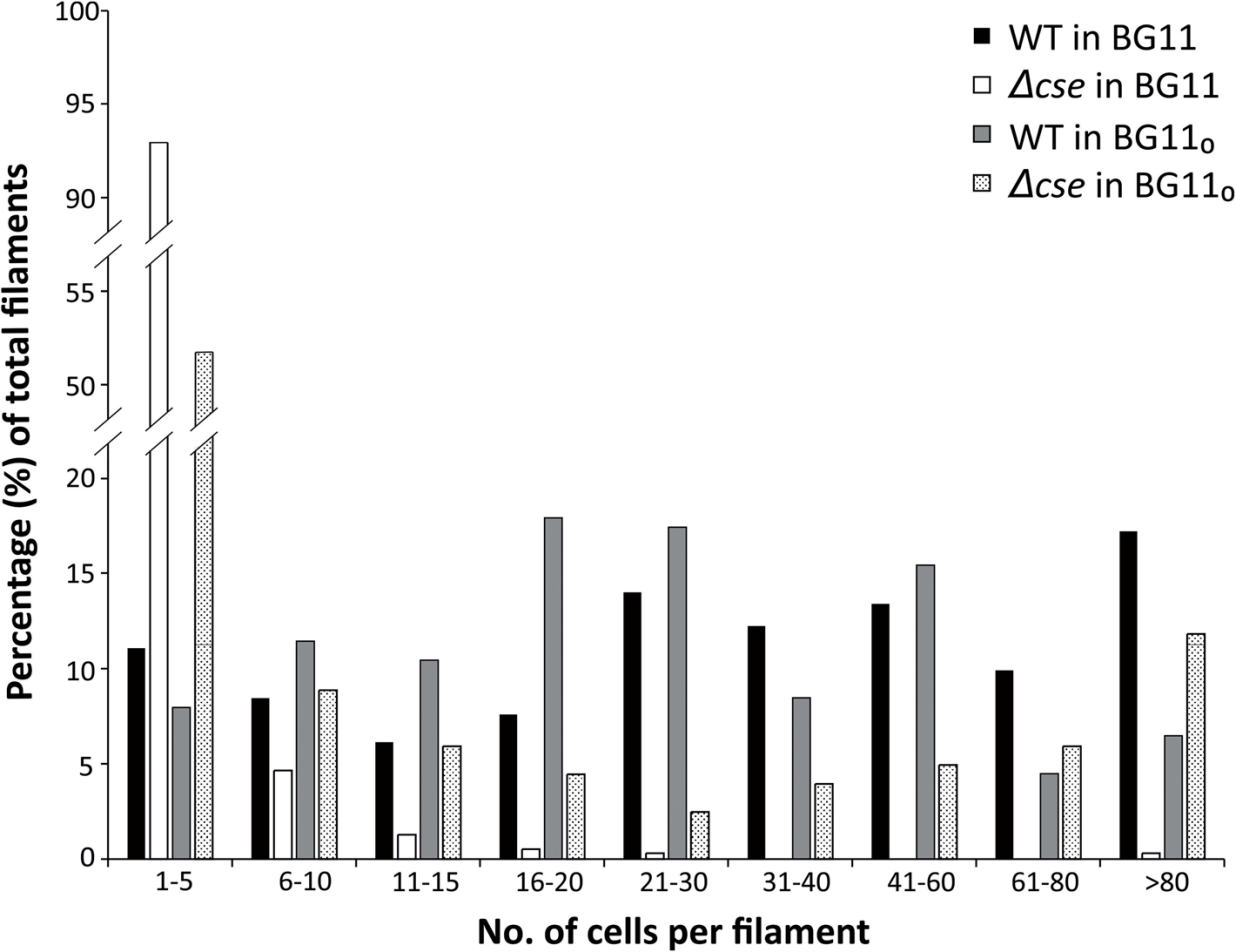
Filament length counts. Range and frequency of filament lengths in wild-type (WT) and *Δcse* cultures grown in BG11 or BG11_0_ in 3% CO_2_, expressed as a proportion of the total number of filaments counted

Two independent *asr1131* deletion clones (*Δcse1* and *Δcse2*) were produced and used to inoculate two separate cultures. Complete segregation of both cultures was confirmed at both DNA and RNA levels (Fig. 1). Both *Δcse* cultures grown in BG11 were composed of predominantly short filaments, compared to the primarily long filaments in the WT strain (Fig. 2a and b). In addition to the short filaments, *Δcse* cultures grown in BG11 in 3% CO_2_ contained a small population of long filaments (Fig. 2b) that resembled WT filaments, including occurrence of heterocyst cells. Growth for 4 days in BG11_0_ medium lacking any combined N source led to an increase in the relative abundance of long filaments containing heterocysts in *Δcse* (Fig. 2d and f), in contrast to the short filaments that were prevalent in BG11 medium. Aggregated clusters of long *Δcse* filaments were harvested from BG11_0_, transferred to either BG11_0_ or BG11 and incubated as previously. After 5 days, short filaments began to appear in the BG11 cultures, and after a further 2 to 3 days, these became the dominant phenotype in the culture, whereas short filaments were detected in BG11_0_ after 10 days (results not shown). Transformation of the *Δcse* mutant with a plasmid expressing *asr1131* under the control of its native promoter abolished the short filament phenotype, with filaments in the complemented strain resembling those in the WT (Fig. 2g). Filament length counts revealed that 93% of the “filaments” in *Δcse* in BG11 comprised only 1–5 cells (Fig. 3), while over 98% of filaments contained less than 20 cells. Only 0.5% of filaments in *Δcse* had more than 80 cells. In contrast, about 70% of WT filaments constituted > 20 cells and 17% comprised > 80 cells in BG11 medium. Under N-fixing conditions (BG11_0_ medium), the majority of WT filaments comprised 16–30 cells, while the proportion of *Δcse* filaments comprising 1–5 cells decreased in BG11_0_ to 52% of total filaments. Conversely, long filaments were more abundant in *Δcse* cultures growing in BG11_0_, in comparison to BG11 medium (Fig. 3).

**Fig. 4:**
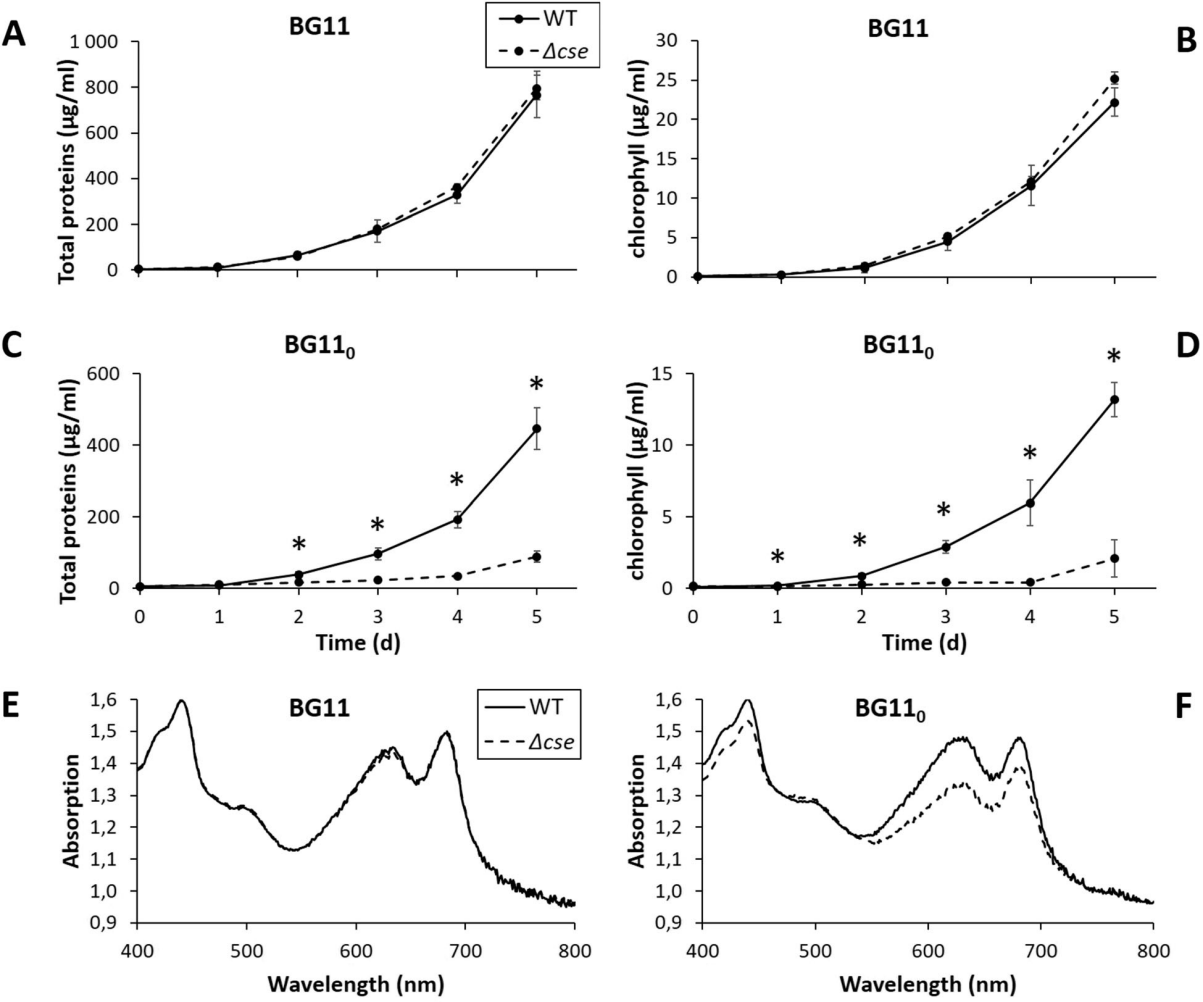
Growth phenotype of the *Δcse* mutant under different growth conditions. Growth curves **a**-**d** of wild-type (WT) and *Δcse* cultures monitored using total proteins (**a** and **c**) and chlorophyll concentrations (**b** and **d**) under 3% CO_2_. Cultures were grown either in regular BG11 medium (**a** and **b**), or in BG11_0_ medium lacking combined nitrogen (**c** and **d**). Data points represent mean values from three biological replicates (*n* = 3), error bars show standard deviations. Significant differences between WT and *Δcse* samples are indicated with asterisks (*t*-test *P* <  0.05). Absorption spectra of WT and *Δcse* cultures were recorded after growing cultures for 5 days in BG11 (**e**) or BG11_0_ (**f**)

The *Δcse* mutant strains were grown alongside WT in BG11 growth media, as well as in BG11_0_ lacking any combined N source (Fig. 4). Protein, chlorophyll and total pigment contents were equivalent in both strains after 5 days in BG11 (Fig. 4a, b and e). In contrast, *Δcse* cultures had very poor growth rates in BG11_0_, compared to the WT (Fig. 4c and d), and developed a yellow colouration after 2 to 3 days that corresponded with decreased contents of chlorophyll (peak at 680 nm) and phycocyanin (peak at 635 nm) relative to absorption at wavelength 750 nm (Fig. 4e and f).

**Fig. 5:**
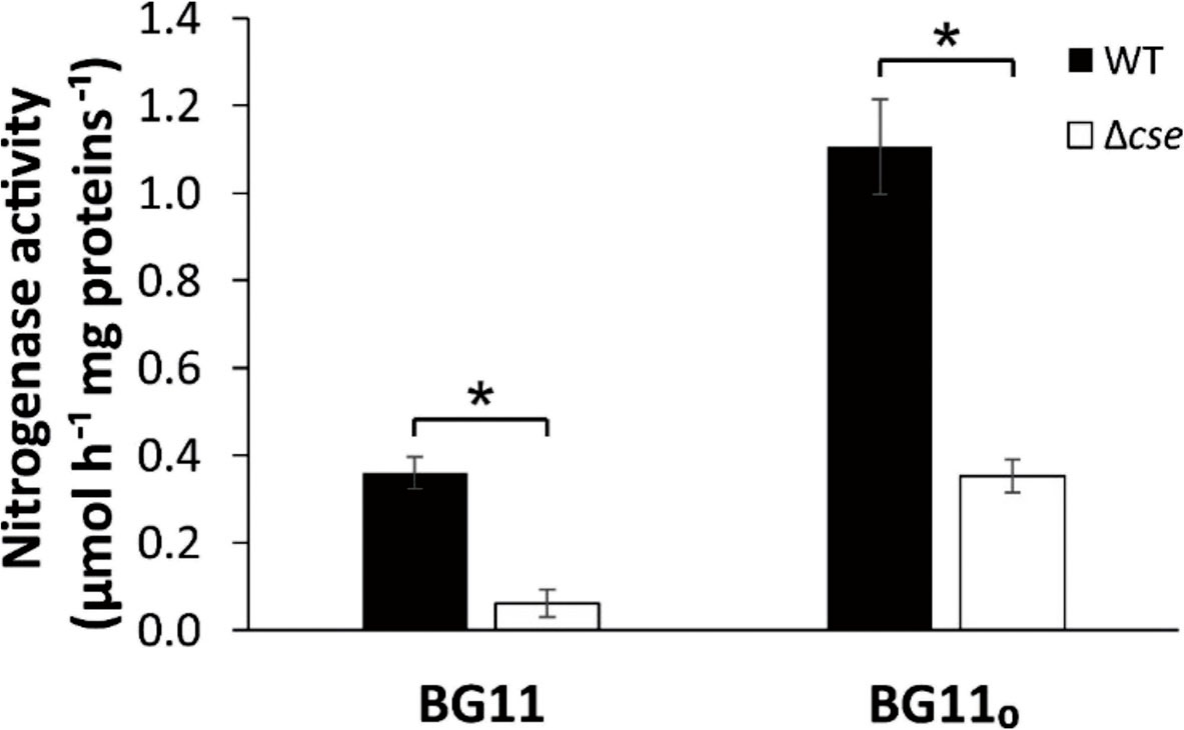
Acetylene reduction assay. Nitrogenase activities of wild-type (WT) and *Δcse* cultures grown for 2 days in BG11 or BG11_0_ in 3% CO_2_. Significant differences between WT and *Δcse* samples (*n* = 3) are indicated with asterisks (*t*-test *P* <  0.05)

Nitrogenase activity was assayed by the conversion of acetylene to ethylene. In N-replete medium, nitrogenase activity in *Δcse* cultures was barely detectable at around 6-fold less than the WT (Fig. 5). After 48 h in N-fixing conditions, nitrogenase activity increased from 0.36 to 1.11 μmol h^− 1^ mg proteins^− 1^ in WT and 0.06 to 0.35 μmol h^− 1^ mg proteins^− 1^ in *Δcse*, with *Δcse* demonstrating around 3-fold lower activity than that of WT in N-fixing conditions.

### Particles adhere to the outer surface of *Δcse* cells

**Fig. 6:**
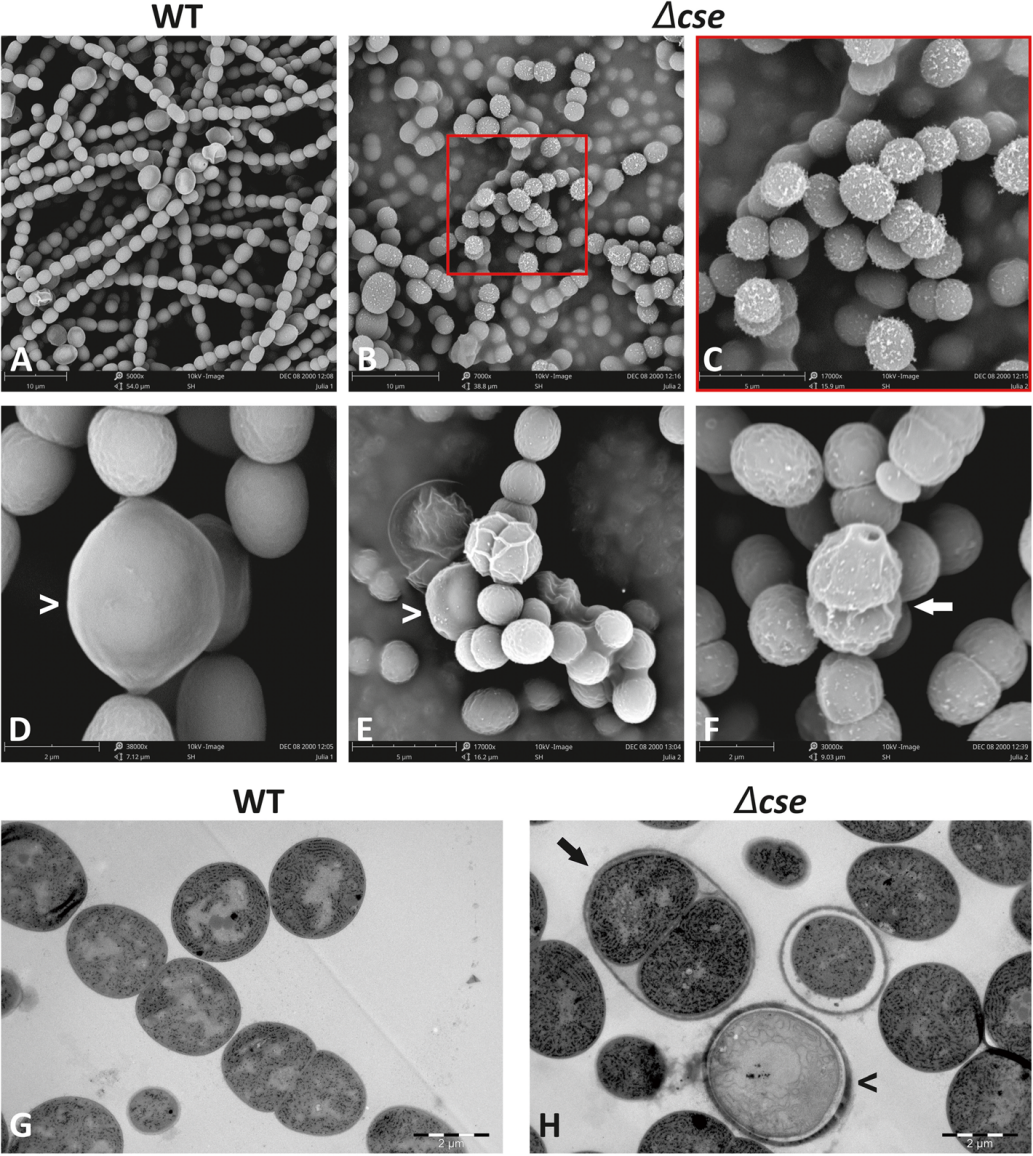
Phenotypic features of the *Δcse* mutant. Electron micrographs of wild-type (WT; left) and *Δcse* (right) cells grown in BG11 medium in 3% CO_2_. **a**-**f** Scanning electron microscopy (SEM) images. **g**-**h** Transmission electron microscopy (TEM) images. Normal heterocysts (carets) and partially-differentiated proheterocysts (arrows) are indicated in (**d**-**h**)

SEM micrographs of WT and *Δcse* cultures highlighted the filament fragmentation phenotype of the mutant (Fig. 6a and b), and also revealed the occurrence of disorganised clumps of *Δcse* cells that appeared to be surrounded by an extracellular membrane or matrix (Fig. 6e). Another striking feature of *Δcse* apparent in the SEM images was the presence of particles of an unidentified substance on the exterior surface of the cells, which was in contrast to the smooth cell surface of WT vegetative cells (Fig. 6a-c). SEM and TEM micrographs revealed the frequent occurrence in *Δcse* cultures of two vegetative cells, which appeared to be newly divided, encapsulated in a mutual outer layer that resembled a heterocyst envelope (see arrows in Fig. 6f and h). Staining of these dividing cells with Alcian Blue confirmed the presence of heterocyst-specific polysaccharides (see Additional Fig. 1).

### *Δcse* has higher concentrations of total sugars

**Fig. 7:**
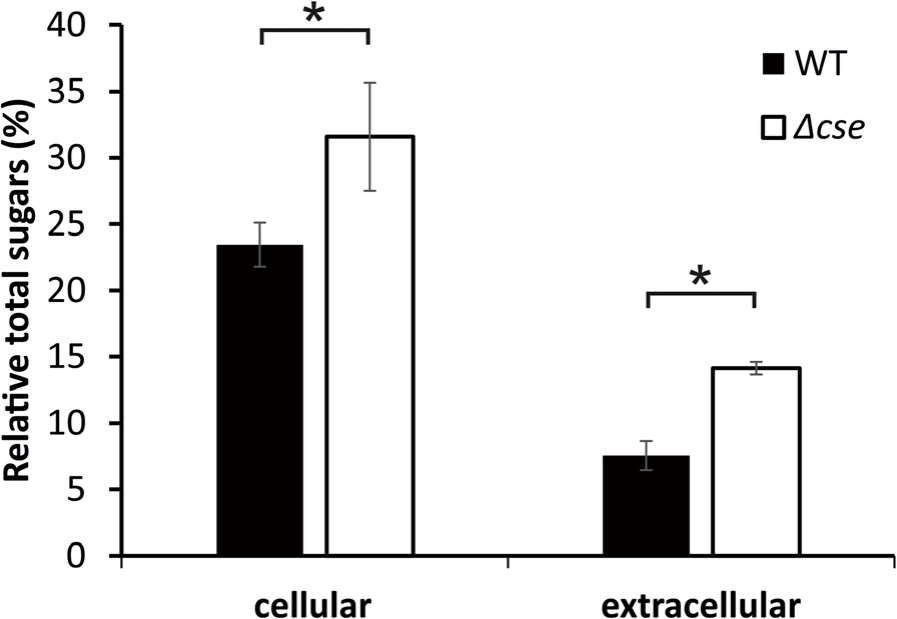
Distribution of accumulated and secreted carbohydrate hexoses of cultures. Total sugars measurements in wild-type (WT) and *Δcse* cell pellets (cellular) and their respective supernatants (growth media; extracellular) relative to the dry biomass. Error bars indicate the standard deviation of three biological replicates (*n* = 3) grown in BG11 in 3% CO_2_. Significant differences between WT and *Δcse* samples are indicated with asterisks (*t*-test *P* <  0.05)

Total sugars were measured in WT and *Δcse* cell pellets, and in the supernatant fractions obtained after centrifugation of cultures grown in BG11. These analyses showed that *Δcse* cells comprised 32% sugars in comparison to 23% in WT cells, relative to their dry biomass (Fig. 7). The total sugars content of the growth media of *Δcse* cultures was approximately twice that isolated from WT growth media (14 and 8%, respectively, relative to the dry biomass).

### Heterocyst differentiation is downregulated at the transcript level

Comparison of the transcriptomes of the *Δcse* mutant strain and the WT grown in BG11 revealed that a majority of differentially expressed genes were related to the formation and function of heterocyst cells. Among the strongest upregulated genes in *Δcse* were *patU5/3*, *asr1734*, *patS*, *hetZ*, *hetP*, *patA*, which are involved in the regulation of heterocyst differentiation (see Table 1). Other heterocyst regulators *ntcA* and *hetC* were only slightly downregulated (FC = − 1.3). Expression of factors responsible for the biosynthesis of the heterocyst envelope and cytoplasmic differentiation was predominantly downregulated. For instance, gene cluster *alr2822* - *alr2841*, which is called the “Heterocyst Envelope Polysaccharide (HEP) island” [38], was downregulated 2 to 3.5-fold, compared to the WT, while gene cluster *all5341* - *all5359*, encoding proteins for glycolipid biosynthesis, was even more strongly downregulated (see Table 1). Genes encoding heterocyst-specific proteins such as flavodiiron proteins (*flv1B/3B*), ferredoxin *(fdxH*), both cytochrome *c* oxidase operons (*cox2/3*), the *devBCA* transporter, *patB* and the uptake hydrogenase cluster were also strongly downregulated. The expression of *nif* genes was mildly to highly repressed (1.1 to 6.5-fold), with *nifH*, *nifK*, *nifB*, *nifS* and *nifW* being the most downregulated. Other clusters associated with N fixation such as *all1424* – *all1427* and *all1431* – *all1440* were also strongly downregulated.

**Table 1:** Transcription changes in the *Δcse* mutant

Accession	Gene symbol	Description	Fold change	FDR
Heterocyst-related genes				
*asr0098*		unknown protein	2.1	0.049
*alr0099*	*hetZ*	heterocyst differentiation	4.1	0.018
*asr0100*	*patU5*	heterocyst inhibitor	3.5	5.69E-06
*alr0101*	*patU3*	heterocyst inhibitor	3.4	4.04E-05
*all0521*	*patA*	two-component response regulator, heterocyst pattern formation protein	2.6	0.002
*asr1734*		heterocyst inhibitor	1.9	0.002
*asl2301*	*patS*	heterocyst-inhibiting signalling peptide	2.7	1.82E-06
*alr2817*	*hetC*	heterocyst differentiation protein	−1.3	0.009
*alr2818*	*hetP*	heterocyst differentiation protein	2.3	0.036
*alr4392*	*ntcA*	nitrogen-responsive regulatory protein	−1.3	0.007
*alr2822 – alr2841*	*hep*	Heterocyst Envelope Polysaccharide island	−2.5	< 0.016
*alr0267*	*hesF*	exoprotein for filament adhesion	−4.8	3.42E-04
*all5341 – all5359*	*hgl, hgd, het*	glycolipid biosynthesis: glycosyltransferases, hgdA-C, hglA-G/T, hetN/I	−4.5	< 0.034
*all2512*	*patB*	heterocyst-specific transcriptional regulator	−4.7	0.026
*all0177*	*flv1B*	heterocyst-specific flavodiiron protein	−3.7	5.19E-09
*all0178*	*flv3B*	heterocyst-specific flavodiiron protein	−3.1	0.034
*all1430*	*fdxH*	heterocyst ferredoxin	−5.5	6.44E-07
*alr2514*	*cox2B*	cytochrome c oxidase 2 subunit II	−3.9	0.003
*alr2515*	*cox2A*	cytochrome c oxidase 2 subunit I	−4.0	0.003
*alr2516*	*cox2C*	cytochrome c oxidase 2 subunit III	−4.4	0.032
*alr2729*		putative membrane protein	−4.3	2.70E-09
*alr2730*		putative membrane protein	−3.8	0.001
*alr2731*	*cox3B*	cytochrome *c* oxidase 3 subunit II	−4.2	2.70E-09
*alr2732*	*cox3A*	cytochrome *c* oxidase 3 subunit I	−5.1	1.73E-04
*alr3710*	*devB*	heterocyst-specific ABC-transporter, membrane fusion protein	−4.0	3.04E-06
*alr3711*	*devC*	heterocyst-specific ABC-transporter, membrane spanning subunit	−3.2	0.009
*alr3712*	*devA*	heterocyst-specific ABC-transporter, ATP-binding subunit	−3.7	0.007
Nitrogen fixation				
*alr1407*	*nifV1*	homocitrate synthase	−3.8	8.36E-05
*asr1408*	*nifZ*	iron-sulfur cofactor synthesis protein	−4.0	0.005
*asr1409*	*nifT*	nitrogen fixation protein	−3.6	0.003
*all1424 – all1427*		nitroreductase family protein, ankyrin, CBS domain containing membrane protein	−3.1	< 0.016
*all1431 – all1440*	*hes & nif*	iron-sulfur cluster biosynthesis protein hesA/B, nifW/X/N/E/K	−5.4	< 0.038
*all1455*	*nifH*	nitrogenase iron protein	−6.5	4.06E-06
*all1456*	*nifU*	nitrogen fixation protein	−4.2	0.009
*all1457*	*nifS*	nitrogenase cofactor synthesis protein	−6.0	0.025
*all1517*	*nifB*	nitrogen fixation protein	−6.4	7.81E-06
*alr2520*		nitrogenase-associated protein	−4.6	7.18E-05
*Hydrogenases*				
*all0688 – alr0700*	*hup*	uptake hydrogenase	−2.7	< 0.009
*alr0766*	*hoxH*	bidirectional hydrogenase large subunit	5.9	0.050
Chlorophyll + pyrimidine biosynthesis				
*all1357*	*hemF2*	coproporphyrinogen III oxidase	17.9	0.011
*alr1358*		Magnesium-protoporphyrin IX monomethyl ester [oxidative] cyclase 1	4.2	2.73E-04
*alr1912*		dihydroorotate dehydrogenase (fumarate)	6.5	0.024
Other gene clusters				
*all2126*			−4.0	2.06E-07
*all2127*		radical S-adenosyl-L-methionine	−6.2	1.52E-04
*all2128*			−7.2	0.001
*alr2522 – alr2527*		unknown proteins, luciferase-alpha subunit	−4.1	< 0.005
*all7191 – all7223*		AIPR protein, ABC transporter, plasmid recombinant protein, ATPase, restriction endonuclease, integrase/recombinase, similar to TrsK protein, two-component response regulator	3.2	< 0.050

Fold change (FC) values indicate differential expression of three biological replicates (*n* = 3) of *Δcse* compared to wild-type (WT) grown in BG11 medium in 3% CO_2_. Genes with FC values ≥1.9 (upregulated) or ≤ −1.9 (downregulated) are shown. In some cases, genes of special interest with FC < 1.9 have been included. False discovery rates (FDR) show *P* values after correction using the Benjamini-Hochberg method. Where operons included > 4 genes, the average FC and largest FDR values are provided

Among the most strongly upregulated genes in *Δcse* were enzymes of the chlorophyll and pyrimidine biosynthesis pathways, including coproporphyrinogen III oxidase (FC = 17.9), dihydroorotate dehydrogenase (FC = 6.5) and the magnesium-protoporphyrin IX monomethyl ester [oxidative] cyclase 1 (FC = 4.2). The bidirectional hydrogenase complex subunit *hoxH* was also upregulated 5.9-fold.

### Knockout of the *cse* gene affects expression of N-regulated genes

**Fig. 8:**
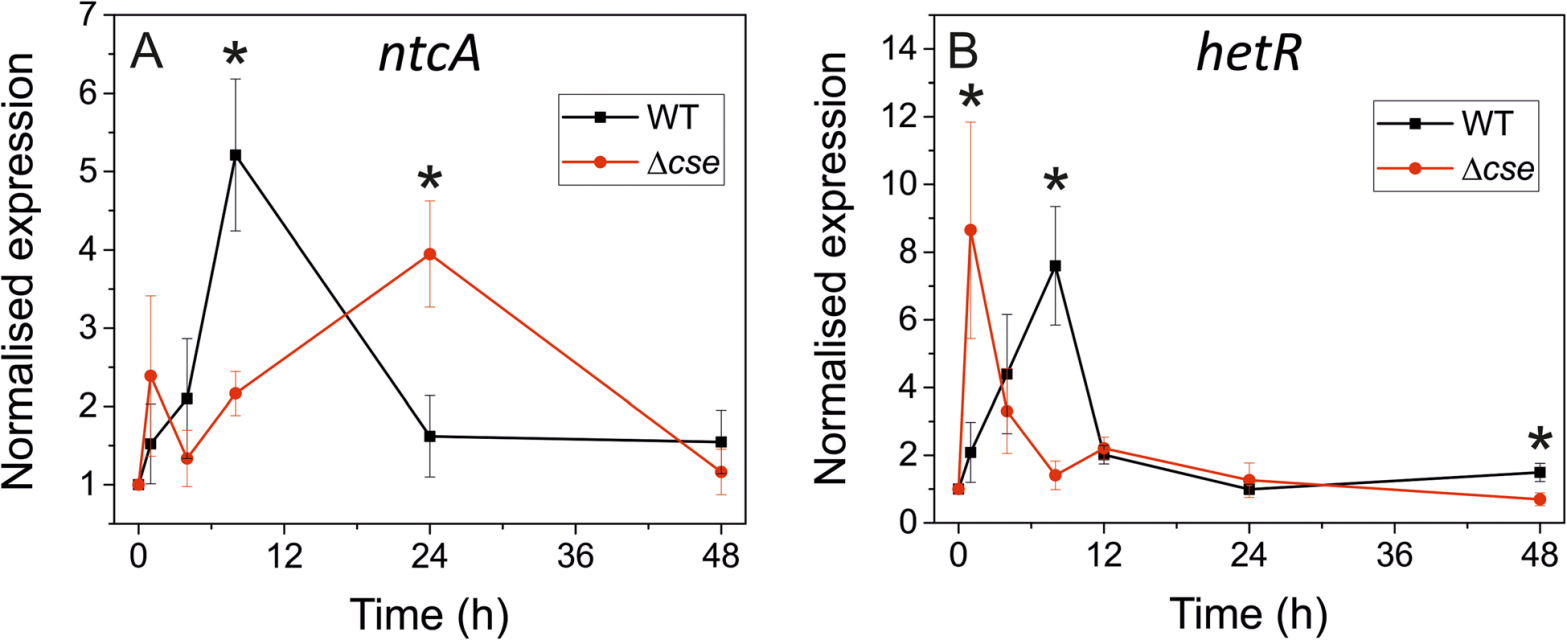
Expression of heterocyst differentiation regulator genes during nitrogen step-down. Expression of *ntcA* (**a**) and *hetR* (**b**) in wild-type (WT) and *Δcse* after nitrogen step-down. Cultures were grown in BG11 medium in 3% CO_2_ and then refreshed in BG11_0_ medium lacking any source of combined nitrogen for a nitrogen step-down. RNA samples were taken at the timepoints indicated. Gene expression values were normalised to the reference gene *rpoA* and the timepoint 0 h values. Error bars indicate the standard deviation of mean values from three biological replicates (*n* = 3). Significant differences between WT and *Δcse* gene expression are indicated with asterisks (*t*-test *P* <  0.05)

To investigate the impact of CSE on the expression of genes involved in heterocyst differentiation, we analysed the expression of *ntcA* and *hetR* genes in WT and *Δcse* during the course of N step-down. In WT, *ntcA* and *hetR* expression peaked after 8 h in N-deficient media, and afterwards returned to pretreatment levels. In *Δcse,* however, *ntcA* expression peaked at 24 h, while *hetR* expression peaked 1 h after the N shift (Fig. 8).

## Discussion

CSE is a small Ca^2+^-binding EF-hand protein, which undergoes a conformational change upon binding of Ca^2+^ [7]. This is a typical feature of Ca^2+^ sensor proteins for the interaction with protein partners, thus translating a Ca^2+^ signal into a physiological response [39]. In *Anabaena*, CSE is the only known Ca^2+^ sensor [7], putatively containing two Ca^2+^-binding EF-hand domains of low and high affinity, similar to calcineurin B, the regulatory subunit of the mammalian serine/threonine protein phosphatase calcineurin A [7, 40–44]. It was shown that *cse* expression responds to changes in the intracellular C/N balance, which is an indicator of the metabolic status of the cell. Expression of *cse* was strongly downregulated 1 h after the shift to N-depleted media, while conversely a low C/N ratio caused a dramatic increase in *cse* expression. Downregulated photosynthetic activity under increased CSE abundance indicated that CSE and Ca^2+^ signalling may link photosynthetic activity with the metabolic status of the cell [7]. In the current study, CSE knockout mutants displayed a striking phenotype of predominantly very short filaments comprising up to five cells, which was restored to the WT phenotype when the *Δcse* mutant was complemented with the *asr1131* gene (Fig. 2g). Several independently conjugated *Δcse* clones were created, and in every case the short filament phenotype came to dominate the culture, although some variation was observed in development of the phenotype over time, ranging from days to weeks. Despite full segregation of *Δcse* cultures, a small proportion of WT-like long filaments persisted. These heterocyst-containing filaments supported the growth of *Δcse* cultures in N-deficient conditions, albeit more slowly and with lower nitrogenase activity than WT cultures and substantial catabolism of N-containing pigments (Figs. 4 and 5). However, the WT-like filaments of the *Δcse* mutant were also susceptible to fragmentation in both N-replete and N-deficient conditions (Fig. 3). These results implicate CSE in a Ca^2+^ signalling pathway that influences filament integrity in *Anabaena*. Filament fragmentation has been previously reported to result from abiotic disturbances, such as osmotic stress through UV radiation, or nutrient deficiencies [45], while high [Ca^2+^] introduced into growth media also induced filament fragmentation [4]. The role of Ca^2+^ signalling in abiotic stress response in *Anabaena* has been demonstrated [1–3], suggesting that fragmentation of *Δcse* mutant filaments may be due to disruption of a stress-responsive Ca^2+^ signalling pathway that leads to an inability to prevent disconnection of filaments. In this case, the WT-like filaments present in *Δcse* cultures may be unstressed cells in which the Ca^2+^ signalling pathway is not activated.

Genes involved in later stages of heterocyst development and nitrogen fixation activity were among the most strongly downregulated in *Δcse* cultures growing in BG11 (Table 1), correlating with a strong decrease in the frequency of heterocysts in these cultures, compared to WT cultures. At the same time, abnormally high expression of *hetP*, *patA*, *patS*, *patU3/5* and other differentiation inhibitors and heterocyst patterning genes in *Δcse* (Table 1) suggested that more *Δcse* cells, compared to WT, commit to enter the “proheterocyst” stage during early stages of heterocyst development, but then fail to develop into mature heterocysts. This indicates that heterocyst maturation was suppressed at the transcriptional level. This may be linked to abnormal activity of the HetR master regulator of heterocyst development due to increased abundance of the PatS peptide in *Δcse*, which is likely to interfere with HetR function [46–48]. Evidence of dysfunctional cell differentiation in the *Δcse* mutant was also apparent in the frequent occurrence of dividing cells enveloped in a heterocyst-like outer layer, revealed by electron and bright-field microscopy (Fig. 6f, h and Additional Fig. 1). These phenomena have been previously identified in short filament mutants of *Anabaena* and are described as partially-differentiated proheterocysts that continued dividing [25, 28, 32, 33], whereas cell division is normally arrested in mature heterocysts [49]. Failure to complete heterocyst commitment and maturation may have been linked to abnormal abundance of differentiation inhibitors described above [50, 51], or interrupted Ca^2+^ signalling during heterocyst development (discussed below). Both NtcA and the signalling molecule 2-oxoglutarate [52] were suggested to control Ca^2+^ signals required for heterocyst differentiation in multicellular cyanobacteria and acclimation to N starvation in unicellular cyanobacteria, defining Ca^2+^ as an early signal of N deprivation [53, 54].

Misdeveloped heterocysts in *Δcse* may have become weak points in the filaments, causing disconnection and disrupting filament integrity. Disrupted dispersion of the inhibitors may have resulted in a higher number of differentiating cells that accumulate inhibitors and thus fail heterocyst maturation, leading to the observed short filament phenotype [51, 55]. Short filament phenotypes similar to that observed in the *Δcse* mutant have been reported in numerous heterocyst formation-impaired mutants [23–31]. The majority of previously reported filament fragmentation mutants have been defective in the Fra proteins [34], of which the septum-localised SepJ/FraG [32, 33] is the most important for filament integrity in N-depleted environments. No significant differences in expression of Fra-encoding genes were observed in our RNAseq data in *Δcse* in comparison to the WT; however, we cannot rule out possible functional interactions between CSE and Fra proteins that may have induced fragmentation in the *Δcse* mutant.

Ca^2+^ signalling plays a vital role in heterocyst differentiation [13], and the evidence presented here suggests that the Ca^2+^-binding CSE protein may be involved in this signalling pathway. Earlier studies have reported a strong and sustained increase in [Ca^2+^]_i_ in *Anabaena* five to six hours after N step-down, which is attributed to downregulation of the Ca^2+^-binding protein CcbP by the master regulators HetR and NtcA [16, 56–60]. However, CSE appears to operate at an earlier point in the heterocyst differentiation process. Indeed, *cse* (*asr1131*) in *Anabaena* WT was included in a group of genes shown to be downregulated by combined N deprivation [61], and *cse* transcription decreased sharply within the first hour of N step-down [7]. This timing appears to correspond with a spike in Ca^2+^ uptake and a small increase in [Ca^2+^]_i_ that have been observed soon after N deprivation in *Anabaena* [13, 16]. This early Ca^2+^ signal of N step-down, which could also arise through uptake of extracellular Ca^2+^ through channels in the plasma membrane [1–3, 62], may activate downstream processes that lead to heterocyst differentiation. In the current work, expression of *hetR* during N step-down was misregulated in *Δcse*, peaking after 1 h compared to 8 h in the WT (Fig. 8b). In addition, HetR- and NtcA-binding sites have been identified in the promoter and terminator region of the *cse* coding sequence [7, 63, 64]. Taken together, these data suggest that CSE may be involved in a Ca^2+^ signalling pathway during the initial stages of heterocyst differentiation, with a decrease in CSE abundance being important for the distinct early transient rise in [Ca^2+^]_i_ that influences the transcriptional regulation activity of HetR [14, 16]. Complete deletion of the *cse* gene would interrupt this early Ca^2+^ signal, leading to unregulated heterocyst development and filament fragmentation.

## Conclusions

CSE appears to be important for Ca^2+^ signalling during changes in cellular C/N balance. In our previous study, downregulation of photosynthesis was linked to CSE upregulation upon C decrease [7], while the current work has implicated CSE in filament integrity and proper cell differentiation in response to low N. The exact mechanism by which the newly discovered CSE protein operates is still not clear, but likely involves a conformational change upon Ca^2+^ binding that induces interaction with an unidentified protein partner. CSE may also regulate the abundance of free Ca^2+^ in the cell, thereby influencing Ca^2+^-sensitive processes like stress response, gene expression and heterocyst development.

## Methods

### Generation of an *asr1131* deletion mutant and complementation

Asr1131 deletion mutant (*Δcse*) strains of *Anabaena* were generated by replacing the 234 bp *asr1131* coding region [65] as well as putative regulatory regions of non-coding DNA 93 and 266 bp up- and downstream, respectively, with a neomycin/kanamycin-resistance cassette (Fig. 1a). 1.5 kb sequences up- and downstream of *asr1131* were amplified from *Anabaena* wild-type (WT) genomic DNA by PCR for homologous recombination in *Anabaena,* using the oligonucleotides *cse*_upst-*Pst*I-S, *cse*_upst-*Xba*I-AS, *cse*_dwst-*BamH*I-S, and *cse*_dwst-*Sal*I-AS (Additional Table 1). The upstream PCR product was digested with *Pst*I and *Xba*I, and the downstream PCR product was digested with *BamH*I and *Sal*I. Both fragments were ligated to a vector containing a neomycin/kanamycin antibiotic cassette and the *sacB* gene for selection of double recombinants obtained from digestion of the plasmids *pRL448* with *Xba*I and *BamH*I, and *pRL271* with *Pst*I and *Sal*I, respectively. The resulting plasmid consisted of a neomycin/kanamycin resistance cassette flanked by *asr1131* upstream and downstream sequences, which was used for triparental conjugation of *Anabaena* WT as described earlier in [66]. Double recombinants were selected on growth media containing 5% sucrose and 200 μg μl^− 1^ neomycin. Full segregation of two independent *Δcse* clones was shown by PCR amplification using the oligonucleotides *cse*_upst-*Pst*I-S and *cse*_dwst-*Sal*I-AS. The *Δcse* mutant strain was complemented by conjugation with *RSF1010*-based replicative vector *pBG2089* expressing *asr1131* under the control of its native promoter and terminator sequences (Additional Table 1). Transformants were selected on media supplemented with 200 μg μl^− 1^ neomycin and 5 μg μl^− 1^ erythromycin.

### Growth conditions and treatments of *Anabaena* and *E.coli* cultures

*Anabaena* WT, *Δcse* and the complemented *Δcse* strain were grown in BG11 [11] buffered with 10 mM TES-KOH (pH 8.0) under constant illumination of 50 μmol photons m^− 2^ s^− 1^ at 30 °C. All cultures were grown in air enriched with 3% CO_2_ with gentle agitation (120 rpm). For the selection of transformants, 40 μg μl^− 1^ neomycin was added to liquid cultures of *Δcse*, and 40 μg μl^− 1^ neomycin and 5 μg μl^− 1^ erythromycin were added to the complemented *Δcse* strain. *E.coli* strains used for cloning were grown in Luria-Bertani (LB) medium supplemented with the antibiotics indicated in the Additional Table 1.

Unless otherwise stated, fresh cultures of *Anabaena* were started at a chlorophyll concentration of 0.1 μg ml^− 1^ in BG11 or BG11_0_ (BG11 lacking NaNO_3_ in the macronutrients, and with CoCl_2_ ∙ 6 H_2_O substituting Co(NO_3_)_2_ ∙ 6 H_2_O in the trace metals). Growth of cultures was monitored over 5 days by measuring total proteins according to a modified Lowry protocol described in [6] and chlorophyll *a* absorption (OD_665_) in 90% methanol. The optical densities and total absorption spectra of cultures were measured with a Thermo Scientific Genesys 10S UV-Vis Spectrophotometer. The dry biomass of cells was determined according to the method described in [6].

### Microscopy techniques

Bright-field images taken with a Zeiss Axiovert 200 M inverted microscope on × 200 magnification were used for filament length counts. At least 200 filaments were counted from each culture grown for 4 days in BG11 starting from OD_750_ = 0.1 under standard growth conditions. Heterocysts were stained with Alcian Blue stain according to the method described in [7].

For Scanning electron microscopy (SEM) and Transmission electron microscopy (TEM), 1 ml of culture grown in BG11 was collected during the exponential growth phase (OD_750_ = 1.0), centrifuged at 1 x g, and the cell pellet fixed in S-collidin buffer and 25% glutaraldehyde (4:1). The samples for SEM and TEM were prepared at the Laboratory of Electron Microscopy (University of Turku, Finland). TEM was performed at the Laboratory of Electron Microscopy (University of Turku, Finland) using a JEM-1400 Plus Transmission Electron Microscope. SEM was carried out at the Institute of Dentistry (University of Turku, Finland). For determination of the percentage of dividing cells, a pair of dividing cells was counted as one cell.

### Determination of total sugars content

Samples of 1 ml of a culture grown for 3 days in BG11 were centrifuged at 6000 x g, and the supernatant and cell pellet were treated separately. After diluting the cell pellets 1:1 with milliQ water in glass tubes, the total amount of sugars in cell pellets and supernatants was determined with a colorimetric method according to [67]. Raw data were normalised to the dry biomass of the culture.

### Nitrogenase activity measurements

Nitrogenase activity of liquid cultures grown in BG11 or BG11_0_ for 2 days was detected using the acetylene reduction assay according to [68], described in [7].

### N step-down experiment and RT-qPCR

WT and *Δcse* strains were grown for 3 days in BG11. During the exponential growth phase (OD_750_ = 1.0), 2 ml samples were frozen for RNA isolation. The cultures were adjusted to OD_750_ = 0.6 in fresh media and shifted to N limited conditions (BG11_0_ medium) after washing once with BG11_0_. Samples of 2 ml were collected and frozen for RNA isolation immediately before (timepoint 0 h) and 1, 4, 8, 12, 24 and 48 h after the shift. RNA isolation was performed as described in [6].

Five hundred nanograms of RNA was utilised for cDNA biosynthesis using the SuperScript III First-Strand Synthesis System (Invitrogen). Transcripts were amplified from 5-fold diluted samples from three biological replicates with the iQ SYBR Green Supermix and the iQ5 Multicolor Real Time PCR Detection System. The reference gene *rpoA* was amplified with the oligonucleotides *rpoA*_qPCR-S and *rpoA*_qPCR-AS. Oligonucleotides used for the analysis of gene expression during N step-down experiments are listed in the Additional Table 1. Normalised expression values were calculated using the Pfaffl method [69].

### RNA isolation and transcriptome sequencing and analysis

WT and *Δcse* strains were grown for 3 days in regular growth conditions in BG11, and 2 ml of culture were taken from three biological replicates (*n* = 3) of each strain during the exponential growth phase (OD_750_ = 1.0). Total RNA was isolated as described in [6], re-extracted in lithium chloride overnight and submitted to the Beijing Genomics Institute (Shenzhen, China) for preparation of single-ended RNAseq libraries and sequencing using Illumina-HiSeq2500. RNAseq reads were aligned to the reference genome of *Nostoc* sp. PCC 7120, downloaded from Ensembl (EBI), using Strand NGS 2.7 software (Agilent, USA). Quantification of the aligned reads was performed using the DESeq R package. Significantly differentially expressed genes were identified by a 2-way ANOVA test using the Benjamini-Hochberg method for false discovery rate (FDR) correction of *P* values.

### Bioinformatics methods

Gene descriptions were obtained from CyanoBase (Kazusa Genome Resources; genome.microbedb.jp/cyanobase), KEGG (www.genome.jp/kegg/), UniProt (www.uniprot.org) and the National Center for Biotechnology Information (NCBI; www.ncbi.nlm.nih.gov).

## Supplementary information

**Additional file 1: Additional Table 1.** List of strains, plasmids and oligonucleotides used in this study. **Additional Figure 1.** Bright-field micrographs of Alcian Blue stained *Anabaena Δcse* filaments. Carets indicate dividing heterocysts.

## Data Availability

The datasets used and/or analysed during the current study are available from the corresponding author on reasonable request.

## Abbreviations

[Ca^2+^]_i_: Intracellular calcium concentration
C: Carbon
Ca^2+^: Calcium ion
CcbP: Cyanobacterial calcium-binding protein
CSE: Calcium sensor EF-hand
FDR: False discovery rate
HEP: Heterocyst envelope polysaccharide
N: Nitrogen
ROS: Reactive oxygen species
SEM: Scanning electron microscopy
TEM: Transmission electron microscopy
WT: Wild-type
