# Supplementary material for: The small Ca2+-binding protein CSE links Ca2+ signalling with nitrogen metabolism and filament integrity in Anabaena sp. PCC 7120
Source: BMC Microbiol. 2020 Mar 11;20:57. doi: 10.1186/s12866-020-01735-5 (PMC7065334; doi:10.1186/s12866-020-01735-5)
Supplement: Supplementary file 1 — Additional file 1: Additional Table 1. List of strains, plasmids and oligonucleotides used in this study. Additional Figure 1. Bright-field micrographs of Alcian Blue stained Anabaena Δcse filaments. Carets indicate dividing heterocysts. [file 12866_2020_1735_MOESM1_ESM.pdf]

**Additional files**

**Additional Table 1** List of strains, plasmids and oligonucleotides used in this study.

| **Strain, plasmid or oligonucleotide** | **Description or sequence** | **Source or reference *** |
|---|---|---|
| ***E. coli* strains** | | |
| BL21 | Recombinant expression of CSE | [1, 2] |
| JM109 | Gene cloning and plasmid construction | [3] |
| HB101 | *Anabaena* conjugation | [4] |
| J53 | *Anabaena* conjugation | [5] |
| ***Anabaena* strains** | | |
| wild-type | *Anabaena* sp. PCC 7120 | [6] |
| *Δcse* | *cse* knockout mutant with neomycin resistance | This study |
| *Δcse::pBG2089* | Complementation of the *cse* knockout mutant with neomycin and erythromycin resistance | This study |
| **Plasmids** | | |
| pET-28a(+) | Histidin-tag and kanamycin resistance | Novagen |
| pET-28a(+)::*cse* | Recombinant expression of CSE with kanamycin resistance | This study |
| *pRL448* | Neomycin/kanamycin resistance | [7] |
| *pRL271* | *sacB* gene from *Bacillus subtilis*, chloramphenicol resistance | [8] |
| *Δcse* plasmid | 1.5 kbp upstream region of *cse* + Nm/Km resistance + 1.5 kbp downstream region of *cse* + *sacB* gene | This study |
| *pBG2089* | low-copy number *asr1131* overexpression vector with erythromycin resistance | [9] |
| *pRL528* | Helper plasmid for bacterial conjugal DNA transfer, chloramphenicol resistance | [10] |
| RP4 | Conjugative plasmid containing an Origin of Transfer (OriT) and transfer genes, ampicillin + kanamycin + tetracyclin resistances | [11, 12] |
| **Oligonucleotides** | | |
| *cse*-NdeI-S | 5’-CCACTCCCATATGGCAACCGAGCAAGAGCTTCAA-3’ | This study |
| *cse*-EcoRI-AS | 5’-GCCGCTGCCAGCGCTGAATTCCTAGGTTAAATTACTTGCTTTCTT-3’ | This study |
| *cse*_upst-*Pst*I-S | 5’-CTGCAGAAAATGGCGATGTCATCTTAGTT-3’ | This study |
| *cse*_upst-*Xba*I-AS | 5’-TCTAGAAAAATTAAGAATACTAAAGGTGA-3’ | This study |

| | | |
|---|---|---|
| *cse*_dwst-*BamH*I-S | 5'-GGATCCAAATGTTCACCAGTTCTAATACT-3' | This study |
| *cse*_dwst-*Sal*I-AS | 5'-GTCGACAAAGCAAATTGGTAGAAGCGCAA-3' | This study |
| *rpoA*_qPCR-S | 5'-CAACTCTCTGTACGGGCCTA-3' | This study |
| *rpoA*_qPCR-AS | 5'-GCTTCTTTCTTGGGGTAAGG-3' | This study |
| *cse*_qPCR-S | 5'-TCAAGACGGCAAAATCTCCA-3' | This study |
| *cse*_qPCR-AS | 5'-CTGTACTAGCAACTCTTGGGG-3' | This study |
| *ntcA*_qPCR-S | 5'-GTCTTCGCGGATTCTACAAA-3' | This study |
| *ntcA*_qPCR-AS | 5'-GCACAAGGAACACCAAAATC-3' | This study |
| *hetR*_qPCR-S | 5'-CCCTGGCAGAGCATATCAAG-3' | This study |
| *hetR*_qPCR-AS | 5'-CCAGTCTTTCATCATGCGGA-3' | This study |

*** References**


1. Studier FW, Moffatt BA. Use of bacteriophage T7 RNA polymerase to direct selective high-level expression of cloned genes. J Mol Biol. 1986;189:113-30.
2. Jeong H, Kim HJ, Lee SJ. Complete genome sequence of *Escherichia coli* strain BL21. Genome Announc. 2015;3:e00134-15.
3. Yanisch-Perron C, Vieira J, Messing J. Improved M13 phage cloning vectors and host strains: nucleotide sequences of the M13mp18 and pUC19 vectors. Gene 1985;33:103-19.
4. Boyer HW, Roulland-Dussoix D. A complementation analysis of the restriction and modification of DNA in *Escherichia coli*. J Mol Biol. 1969;41:459-72.
5. Clowes RC, Rowley D. Some observations on linkage effects in genetic recombination in *Escherichia coli* K-12. J Gen Microbiol. 1954;11:250-60.
6. Rippka R, Deruelles J, Waterbury JB, Herdman M, Stanier, RY. Generic assignments, strain histories and properties of pure cultures of cyanobacteria. J Gen Microbiol. 1979;111:1-61.
7. Elhai J, Wolk CP. A versatile class of positive-selection vectors based on the nonviability of palindrome-containing plasmids that allows cloning into long polylinkers. Gene 1988a;68:119-38.
8. Cai YP, Wolk CP. Use of a conditionally lethal gene in *Anabaena* sp. strain PCC 7120 to select for double recombinants and to entrap insertion sequences. J Bacteriol. 1990;172:3138-45.
9. Walter J, Selim KA, Leganés F, Fernández-Piñas F, Vothknecht UC, Forchhammer K, et al. A novel $Ca^{2+}$-binding protein influences photosynthetic electron transport in *Anabaena* sp. PCC 7120. Biochim Biophys Acta Bioenerg. 2019;1860:519-32.
10. Elhai J, Wolk CP. Conjugal transfer of DNA to cyanobacteria. Methods Enzymol. 1988b;167:747-54.
11. Datta N, Hedges RW, Shaw EJ, Sykes RB, Richmond MH. Properties of an R factor from *Pseudomonas aeruginosa*. J Bacteriol. 1971;108:1244-9.

12. Pansegrau W, Lanka E, Barth PT, Figurski DH, Guiney DG, Haas D, et al. Complete nucleotide sequence of Birmingham IncP alpha plasmids. Compilation and comparative analysis. J Mol Biol. 1994;239:623-63.

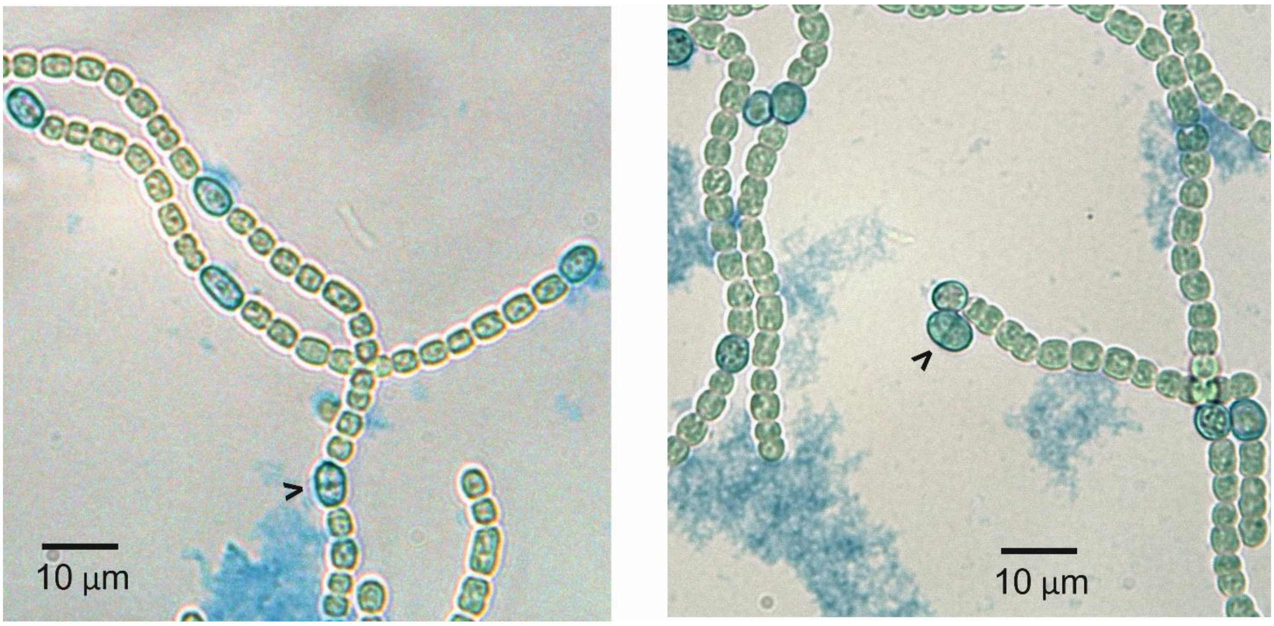


**Additional Fig. 1:** Bright-field micrographs of Alcian Blue stained *Anabaena Δcse* filaments. Carets indicate dividing heterocysts.
